# Effects of High Dietary Inclusion of Defatted Mealworm (*Tenebrio molitor*) Meal as a Fish Meal Substitute on Growth, Histological Traits, and Health Performances of Rainbow Trout (*Oncorhynchus mykiss*)

**DOI:** 10.1155/anu/5568058

**Published:** 2025-01-29

**Authors:** Md. Sakhawat Hossain, Ali Hamidoghli, Jeongwhui Hong, Wendy Sealey, Brian C. Small

**Affiliations:** ^1^Aquaculture Research Institute, Hagerman Fish Culture Experiment Station, University of Idaho, 3059F National Fish Hatchery Road, Hagerman 83332, Idaho, USA; ^2^Department of Aquaculture, Faculty of Fisheries, Sylhet Agricultural University, Sylhet 3100, Bangladesh; ^3^Department of Fisheries Science, Faculty of Aquatic Biology, Chonnam National University, 50 Daehak-ro, Yeosu 59626, Jeonnam, Republic of Korea; ^4^U.S. Department of Agriculture, Agricultural Research Service, 4050 Bridger Canyon Road, Bozeman 59715, Montana, USA; ^5^Department of Fish and Wildlife Sciences, University of Idaho, Moscow 83844, Idaho, USA

**Keywords:** defatted mealworm, digestibility, growth, health, histological traits, rainbow trout

## Abstract

The digestibility of defatted mealworm (DMW, *Tenebrio molitor*) and its substitution for fishmeal (FM) in rainbow trout (*Oncorhynchus mykiss*) diets was determined. In the first trial, a diet with 40% FM was considered as the control (Diet 1), and four other diets replaced 25% (Diet 2), 50% (Diet 3), 75% (Diet 4), and 100% (Diet 5) of FM with DMW. In the second trial, the in vivo digestibility of DMW was assessed. Results of the first trial showed no significant differences in fish growth and feed utilization performance among dietary groups (*p*  > 0.05). Fish fed the highest DMW level showed significantly higher whole-body lipid and energy composition compared to fish fed the control diet. Substitution of FM with DMW did not significantly affect hematological electrolyte, acid–base, and blood gas parameters, except for sodium (Na) concentration. The measured mean score of histopathological features did not show significant degradation of the distal intestine when FM was completely replaced. A significantly lower liver inflammation was observed in fish-fed Diet 3 compared to fish-fed Diet 5. Based on the results of the second experiment, the apparent digestibility coefficient of dry matter, protein, lipid, energy, and phosphorus were 80%, 87%, 100%, 84%, and 90%, respectively, and all essential amino acid digestibility coefficients were >90%. Overall, the highly digestible DMW can fully replace FM in the diet, up to 40% as fed, with little to no impact on the health, growth performance, feed efficiency, and fillet composition. When considering all parameters evaluated, an inclusion level between 20% and 30% (as fed) or 50%–75% of FM replacement was found to yield the best performance and fish health.

## 1. Introduction

With the decline of fishmeal (FM) global supply from its high in the late 1990s, there have been increasing challenges with the availability and costs of this valuable ingredient in aquaculture. Meanwhile, replacing FM in the diet of carnivorous species such as rainbow trout (*Oncorhynchus mykiss*) has been an ongoing concern for the industry. As a result, numerous substitute protein sources have been examined over the past 40 years [1]. Due to their advantageous amino acid composition and availability, soybean meal and soy concentrates have taken the lead [2]. But trout and other salmonids have difficulties digesting diets with high levels of plant proteins. Antinutritional factors such as saponins, trypsin inhibitors, and tannins are substances that interfere with the proper utilization of nutrients [1]. Antinutritional substances and antigens in plant ingredients cause enteritis of the distal intestine, which in turn affects health, growth, and carcass nutrient consumption [3–5]. Even though soy is still a common ingredient in fish feeds today, the demand for alternative protein ingredients has increased due to rising pricing and enteritis caused by soybean meal. The recent demand for sustainable protein alternatives is also fueled by worries about soy's impact on rainforest loss and other sustainability issues.

Insect meal is a relatively new addition to the nutrition toolkit for fish and offers a suitable macronutrient and amino acid composition [6]. Defatted yellow mealworm (also known as darkling beetle; *Tenebrio molitor*) meal contains about 72% protein and 5% lipid. Furthermore, the production of mealworms is promoted as sustainable, with the potential for 365 harvests every year. In comparison to soybean cultivation, mealworm production capability can produce 5000 times the amount of soy per acre while using only half the energy and 2% of the water [7]. Although high in protein, defatted mealworm (DMW) meal can be limiting in methionine, lysine, and threonine for trout, is low in omega-3 fatty acids, and contains considerable amounts of chitin [8, 9].

Despite some limitations, the DMW meal has already been tested as a partial alternative for traditional protein sources for farming several different aquaculture species [10–14]. Depending on the types of mealworm meal (defatted/full-fat meal) and fish species, it is evident that mealworm meal can replace a big portion of FM in aquafeed [8]. For example, in pearl gentian grouper *Epinephelus lanceolatus × Epinephelus fuscoguttatus*, only 12% replacement for FM was achieved without negatively affecting fish growth [15]. In the large yellow croaker *Larimichthys crocea*, Zhang et al. [13] reported replacing 15% FM (6.7% DMW meal inclusion) and showed better humoral immunity and intestinal health without negatively impacting growth. In other studies with gilthead sea bream *Sparus aurata* [10], blackspot sea bream *Pagellus bogaraveo* [16], rainbow trout [17], and large yellow croaker [14], 25%–50% dietary FM replacement was achieved without having any negative impacts on fish growth performance. A relatively higher (50%–70%) FM replacement with dried full-fat mealworm meal was also reported in some fish species, such as gilthead sea bream [10], European sea bass *Dicentrarchus labrax* [18], blackspot sea bream [16], and rainbow trout [17, 19], without any adverse effect on fish growth performance. A total substitution of FM with DMW meal (65% DMW meal inclusion) has been testified in red sea bream *Pagrus major* with significant growth promotion [11]. Chemello et al. [12] evaluated DMW meal in rainbow trout grower diets and stated that DMW meal could entirely substitute FM in commercial rainbow trout diets without having any adverse impacts on the performance of fish. However, it should be noted that these authors used a maximum level of 20% DMW meal to replace 100% of FM. Also, in red sea bream, a total FM replacement with greater incorporation of DMW meal was successful [11]. Given the reported results, research relating to even greater inclusion of DMW meal (>20%) in rainbow trout diets is needed and has not been previously reported.

When investigating a new feed ingredient, in addition to evaluating growth performance and digestibility, gastrointestinal health, hematological and arterial blood characteristics, and morphology of the liver and kidney should also be considered. The intestine serves as the primary site of digestion and nutrient absorption, and intestinal health has a substantial impact on how effectively dietary nutrients are utilized [20, 21]. The liver is one of the most important organs in the metabolism of nutrients [22], and the kidney is a vital osmoregulatory and excretory organ in fish [23]. To ensure the nutritional effectiveness of a new diet, it is imperative to assess the morphological changes that may take place in these vital organs [20, 24]. Researchers have been using histology in conjunction with other physiological indicators to understand the tolerance of fish to novel feed ingredients [21]. Nevertheless, there is a paucity of research on the effects of DMW on the hematological and arterial blood characteristics (electrolyte, acid–base, and blood gas values) and histological traits of the vital organs (liver, kidney, and intestine) in aquatic species. Therefore, the goal of the present research was to assess the digestibility of DMW meal in rainbow trout and evaluate the effects of high inclusion levels as an FM substitute on growth, histological traits of intestine, liver and kidney, and hematological and arterial blood parameters.

## 2. Materials and Methods

### 2.1. Experimental Design and Test Diets

The DMW meal was obtained from Beta Hatch (Cashmere, WA, USA). The nutritional value of the DMW meal utilized in the experimental diets was analyzed for nutrient content at the Midwest Laboratories, Omaha, Nebraska (Table 1). These data were utilized to fine-tune feed formulations and reach desired nutrient levels. As illustrated in Table 2, five experimental rainbow trout diets were produced. A diet with 40% FM was considered as the control (Diet 1), and four other diets replaced 25% (Diet 2), 50% (Diet 3), 75% (Diet 4), and 100% (Diet 5) of FM in the control diet with DMW meal.

At the Bozeman Fish Technology Center (Bozeman, Montana, USA), experimental feeds were extruded into 4.5 mm-diameter pellets under conditions similar to those used in commercial fish feed manufacturing. The experimental ingredients were processed through an air-swept pulverizer (Model 18H, Jacobsen, Minneapolis, MN, USA) to a particle size of 200 µm and then through a twin-screw cooking extruder (DNDL-44, Buhler AG, Uzwil, Switzerland), with a residence time of 25 s to 127°C in the extruder barrel (on average across five sections). Final moisture contents of under 10% were attained by drying pellets in a pulse bed dryer (Buhler AG, Uzwil, Switzerland) for 20 min at 102°C. A cooling time of 10 min was allotted after drying the feed. Then, a vacuum-coater (AJ Mixing, Ontario, CA, USA) was used to top-coat all the added oil. Diets were transported to the Hagerman Fish Culture Experiment Station (HFCES) and stored there until the feeding trial.

### 2.2. Feeding Trial

This study used rainbow trout fingerlings that were hatching from eggs purchased from a commercial source (Trout Lodge, Sumner, WA, USA). To acclimate to the lab environment, fish were kept at HFCES in several 145 L tanks and fed a commercially prepared diet (crude protein 45% Skretting, USA) for 3 weeks. Twenty 145-L tanks were randomly assigned to each of the five diets in quadruplicates and supplied with 40 rainbow trout juveniles (starting weight 23 ± 0.4 g). Each tank received 8 L/min of spring water in a flow-through system that was gravity-fed at a consistent temperature of 15 ± 0.5°C and pH of 7.7 ± 0.1. Fish were hand-fed twice each day to daily satiation for 13 weeks. Electric timers were used to keep the photoperiod at 10 h of darkness and 14 h of light. During the experiment, dead fish were immediately removed from the tanks, and tanks were cleaned daily. The Institutional Animal Care and Use Committee (IACUC) at the University of Idaho authorized the experimental protocol, including fish handling and sampling (IACUC-2021-34).

### 2.3. Fish Sampling

For the initial whole-body composition analysis, 15 fish were chosen at random prior to the feeding trial. According to Bai, Hardy, and Hamidoghli [25], fish from each tank were fasted for 24 h after the feeding trial, counted, and weighed in batches to assess the growth and feed utilization variables, such as specific growth rate, weight gain, feed conversion ratio, and protein efficiency ratio. MS-222 (100 mg L^−1^, buffered to pH 7.0) was used to anesthetize four randomly chosen fish in each tank (*n* = 16 fish per diet) for blood collection. Blood samples were used for the analysis of blood chemistry using a VetScan i-STAT 1 handheld analyzer (Abaxis Products, Union City, CA, USA) for hemoglobin, hematocrit, glucose, ionized calcium, potassium, sodium, acidity (pH), partial pressure of carbon dioxide (pCO_2_), bi carbonate (HCO_3_), base excess (BEcef), total carbon dioxide (TCO_2_), oxygen saturation (sO_2_), and partial pressure of oxygen (PO_2_). Following euthanasia, the fish were dissected to collect the distal intestine, liver, and trunk kidney for histological examination. Segments measuring 0.5–1 cm were removed following the collection of the distal intestines, and the intestinal content was flushed out of the lumen with phosphate-buffered saline (Gibco; pH 7.4). Subsamples of the liver, distal intestine, and trunk kidney utilized for histology were placed in falcon tubes with buffered formalin as the fixative. In preparation for the whole-body nutrient composition assays, four more fish from each tank were collected, euthanized, and put in storage at –20°C.

### 2.4. Histology of Distal Intestine, Liver, and Trunk Kidney

Samples of the distal intestine, trunk kidney, and liver, which had been preserved in buffered formalin, were moved to 50% isopropanol until they were prepared using the standard histological methods employed by the US Fish and Wildlife Service [26]. Histopathological evaluations were performed following staining with hematoxylin and eosin. According to Urán et al. [27], a semi-continuous scoring system (0–5) was used to evaluate mucosal fold fusion (MFF), goblet cell (GC), supranuclear vacuole (SV), eosinophilic granulocyte (EG), lamina propria (LP), and subepithelial mucosa (SeM). These parameters were used to determine the occurrence of distal intestinal enteritis in fish fed different dietary treatments. Ratings of 0–1 indicated normal morphological variation, whereas ratings >2 indicated morphological changes compatible with an intensifying intestinal inflammatory response. All intestinal slides were prepared and randomly scored using light microscopy (Zeiss Axioscope A1, Carl Zeiss Ltd, Cambridge, UK) at the Bozeman Fish Health Center and HFCES, respectively. The Bozeman Fish Health Center also prepared the liver and kidney slides. These slides were then scored at Fishhead Labs LLC using a Nikon Eclipse 80i (Tokyo, Japan), and photomicrographs were taken using an Amscope MU 1403 (Irvine, CA, USA). All slides were evaluated for the presence or absence of lesions. If lesions were present, a short note was documented to describe the change.

### 2.5. Digestibility Trial

After feeding DMW meal to rainbow trout (about 150 g), in vivo digestibility was assessed. A reference diet (Table 3) was made up of practical ingredients and 0.1% of an indigestible inert marker (yttrium oxide). From this, a test diet was made up of 30% of the test ingredient and 70% of the reference diet on a dry-matter basis. After 2 weeks of feeding the test and reference diets, the fish in each tank were sedated with the use of MS-222. They were then taken out of the water for 20–30 s, and the procedure known as stripping was used to gently collect their fecal matter into aluminum pans. To get enough fecal samples, fish were stripped twice, 4 days apart [25]. In between stripping collections, feces were kept frozen at –20°C. Feces samples were dried in were dried in a convection oven at 70°C for 24 h. Analysis of proximate composition, energy, mineral, and amino acid content was performed on samples of DMW ingredients, diet, and respective feces. The apparent nutrient digestibility coefficient (ADC) for energy, amino acids, and macronutrient digestibility were determined using equations explained below [25]:

  
$$\begin{array}{c} \text{ADC (\%)}=100-\left(100\times \left({\text{\% Y}}_{2}{\text{O}}_{3}\text{ in diet}/{\text{\% Y}}_{2}{\text{O}}_{3}\text{ in feces}\right)\times \left(\text{\% nutrient in feces/\% nutrient in diet}\right)\right), \end{array}$$

  
$$\begin{array}{c} \text{ADC of DMW meal (\%)}=100/30\;\left[\text{(nutrient digestibility of the test diet)}–70/100\text{(nutrient digestibility of the reference diet)}\right]. \end{array}$$

### 2.6. Biochemical Analysis

The HFCES lab conducted analyses on fish whole-body and feed samples to determine their nutritional composition and gross energy. These analyses followed standard protocols and were performed in duplicates. Proximate composition, which includes moisture, protein, fat, and ash content, was determined using procedures outlined in AOAC [28]. A bomb calorimeter (Parr 6300, Parr Instrument Company Inc., Moline, IL, USA) was used to determine the energy content in samples. An amino acid analyzer was used at Midwest Laboratories in Missouri, USA, to measure the amino acids in experimental fish feed and excrement. Inductively coupled plasma (ICP; AOAC 985.01) was used to analyze minerals, including yttrium, in the Department of Agricultural Chemistry, Louisiana State University Agricultural Center, Baton Rouge, Louisiana. Duplicate analyses of each were performed. Using test cartridges (Abaxis products, Union City, CA, USA), hematocrit, hemoglobin, glucose, sodium, potassium, pH, ionized calcium, pCO_2_, HCO_3_, base excess, TCO_2_, sO_2_, and PO_2_ were analyzed using a VetScan i-STAT 1 handheld analyzer.

### 2.7. Statistical Analysis

Tank mean values were used as the observational units in the statistical study. The data for fish husbandry variables, body composition, hematological and arterial blood parameters, and histology scores were checked for normality and homogeneity of variance. In cases where the standard assumptions were satisfied, the data underwent statistical analysis by one-way analysis of variance (ANOVA) employing the general linear model. Polynomial orthogonal contrast was then employed to ascertain the linear and quadratic trends of the parameters in relation to the dietary inclusion of DMW meals. If significant differences were discovered, the data were subjected to Tukey's HSD test to separate the means using the IBM SPSS version 22 tool, with a significance level of *p* < 0.05.

## 3. Results

### 3.1. Experimental Diets Nutritional Composition

The proximate composition analysis of experimental diets indicated that all diets were isonitrogenous and isoenergetic (Table 4). Also, amino acid analysis showed approximately similar profiles across the experimental diets. The analyzed nutrients in diets satisfied or exceeded rainbow trout's nutritional needs [29].

### 3.2. Growth Performance and Feed Utilization

After 13 weeks, rainbow trout in all treatment groups grew close to 11 times their starting weight, with an average final body weight ranging from 242.4 to 268.3 g (Table 5). Fish growth and feed use, however, showed no statistically significant differences between treatments and neither linear nor quadratic trend was observed. The addition of DMW instead of FM had no discernible impact on feed consumption. Fish survival (%) ranged from 86.9% to 96.9%, and dietary treatments did not significantly differ from one another. Fish fed the various diets did not exhibit any significant differences and neither linear nor quadratic trend was observed in the measured somatic indices like hepatosomatic index (HSI) and condition factor (CF).

### 3.3. DMW Meal Digestibility

Table 6 lists the apparent digestibility coefficients for DMW meals. DMW meal amino acid digestibility coefficients were mostly over 90%, except for cysteine (79.30%) and taurine (72.34%).

### 3.4. Proximate Compositions of the Whole-Body and Fillet

The proximate compositions of key nutrients in juvenile rainbow trout were examined at the beginning and end of the study, as indicated in Table 7. All fish exhibited alterations in the assessed variables, including moisture, protein, lipid, and energy, when related to their initial levels. Fish given Diet 1 had notably higher whole-body moisture content, while those on Diet 5 had lower levels, and the other groups displayed intermediate values. Also, whole-body moisture content was found to follow a linear trend. Fish-fed Diet 4 and Diet 5 had considerably greater and lower levels of whole-body protein, respectively, whereas other groups had intermediate levels. However, whole-body protein neither followed a linear nor quadratic trend. Fish-fed diets containing DMW had linearly increased whole-body lipid and energy contents in contrast to the control (Diet 1) group, with the significantly highest value representing fish-fed Diet 5. Dietary groups had no discernible impact on the total body ash content. The proximate composition of the major nutrients and the energy content of the final harvested fish fillets remained unaffected by the dietary treatments, as presented in Table 7.

### 3.5. Hematological and Arterial Blood Characteristics

Table 8 shows the hematological and arterial blood characteristics (blood gas and biochemical parameters) of rainbow trout given trial feeds for 13 weeks. All the measured hematological and arterial blood features (hematocrit, hemoglobin, glucose, ionized calcium, potassium, sodium, pH, HCO_3_, pCO_2_, PO_2_, TCO_2_, base excess, and sO_2_) remained unaffected by the dietary treatment except sodium. Sodium content tended to increase linearly with the increasing dietary DMW levels but exhibited no quadratic effects (*p*  > 0.05). Fish given Diet 5 exhibited significantly higher sodium content, followed by Diet 3; other groups showed significantly lower sodium content.

### 3.6. Histopathology of Distal Intestine, Liver, and Kidney

#### 3.6.1. Distal Intestinal Histopathology

**Figure 1:**
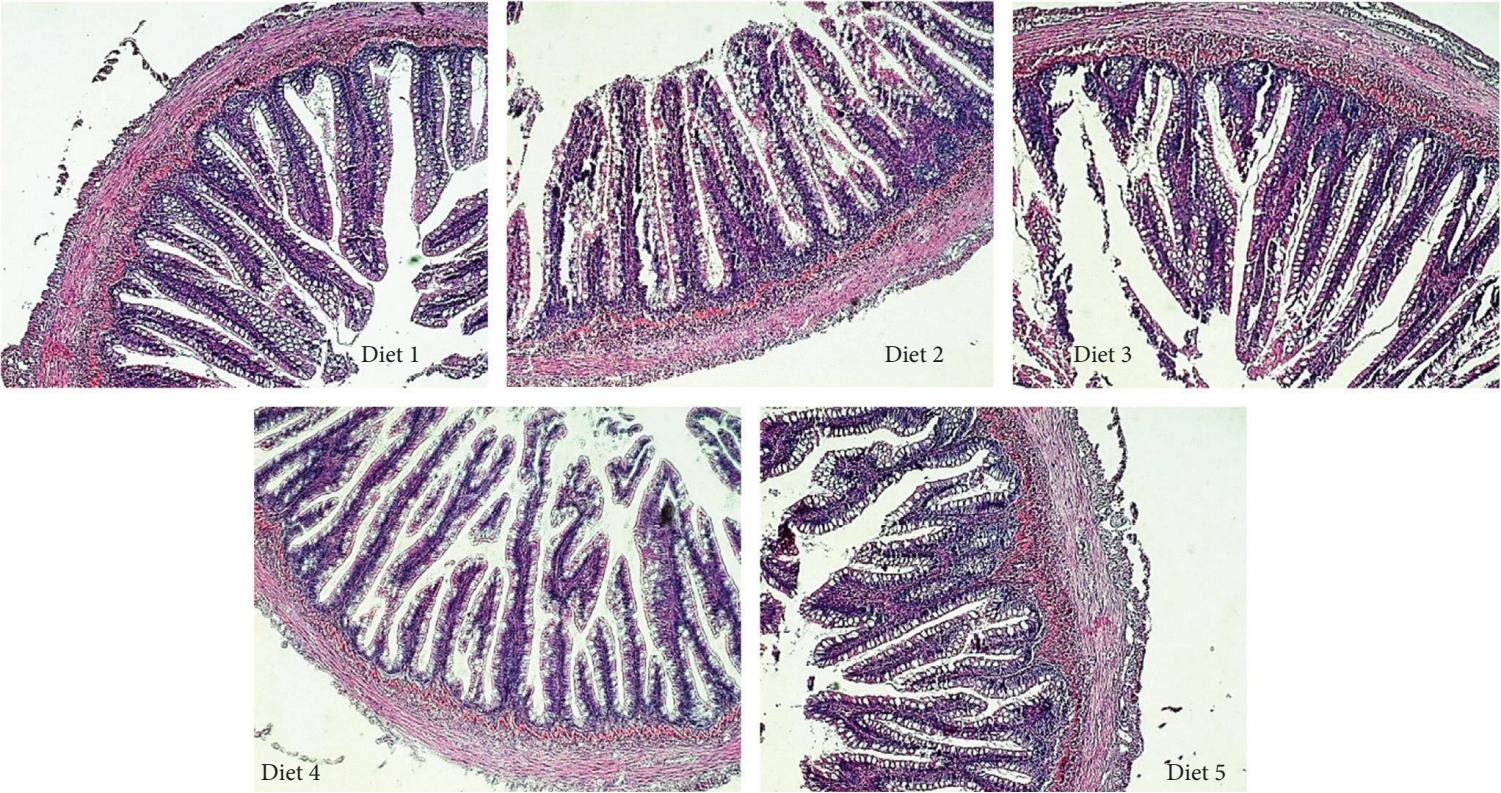
Photomicrographs (H&E, 10× magnification) of representative histological sections showing the distal intestinal morphology of rainbow trout fed experimental Diets 1–5 for 12 weeks.

Representative histological sections from each dietary treatment group are shown in Figure 1.  Table 9 presents the distal intestinal histopathology scoring of rainbow trout-fed experimental diets for 13 weeks. According to the results, only LP thickness was significantly influenced by the dietary treatments and followed a linear trend. The dietary treatments had no significant impact on other examined characteristics (GCs, MFF, EG, SV, and SeM). Fish-fed control and Diet 5 demonstrated a lower and higher mean histopathological score, respectively, which is an indicator of gross distal intestine enteritis. However, statistically, there were no significant differences.

#### 3.6.2. Liver Histopathology

**Figure 2:**
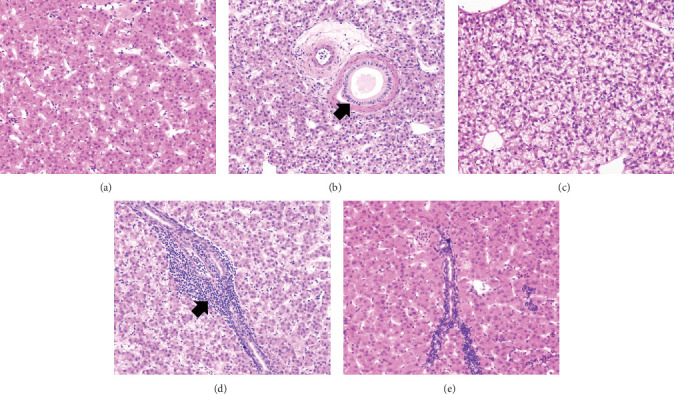
Photomicrographs (H&E, 20× magnification) of normal hepatocytes (a) and normal portal area with a bile duct (arrow), artery, and vein (b) representative of Diets 2–3. Glycogen-type vacuolar change, where hepatocytes have wispy indistinct intracytoplasmic vacuoles can be seen in this photomicrograph of a fish-fed Diet 4 (c), and lymphocytic inflammation can be observed surrounding a bile duct as seen in this photomicrograph of a of fish-fed Diet 5 (d) (arrow). Fish-fed Diet 1 (e) was observed to be an intermediary to Diets 4 and 5 with observations of lymphocytic inflammation.

**Figure 3:**
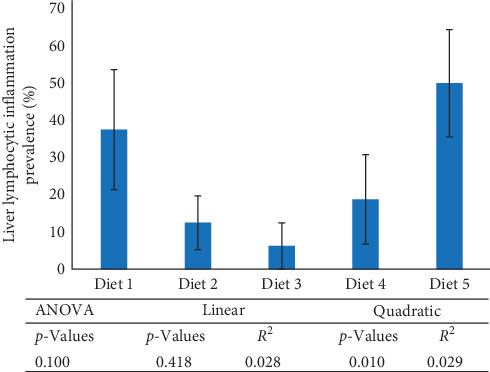
Liver lymphocytic inflammation prevalence (%) of rainbow trout fed experimental diets for 13 weeks. Data represent means ± SEM (*n* = 16 fish per treatment). ANOVA, analysis of variance.

The general conformation of the liver for the rainbow trout was within normal limits (Figure 2). The liver was composed of organized cords of polygonal hepatocytes that are two cells wide. Separating cords were sinusoidal vessels lined by attenuated endothelium. Evenly scattered throughout the parenchyma were portal areas containing a bile duct, artery, and vein supported by fibrocollagenous connective tissue. Furthermore, larger caliber veins were also distributed in a normal pattern throughout the tissue. Hepatocytes had minimal variation in cellular size and shape, but there was mild variation in cytoplasmic contents. Intracytoplasmic vacuolar change characterized as lipid-type or glycogen-type was seen in a subset of fish from all groups (Figure 2). Minimal-to-mild pathological changes in the form of rare lymphocytic aggregates (Figure 2) were randomly scattered throughout the hepatocellular cords or expanding the connective tissue in the portal in all five diet treatments. Fish-fed Diets 2–4 had a lower prevalence of lymphocytic inflammation (%), with Diet 3 fish having the least prevalence. In contrast, fish-fed Diet 5 had the highest prevalence of lymphocytic inflammation (Figure 3). Across treatments, liver lymphocytic inflammation prevalence (%) followed a quadratic trend and not a linear trend (Figure 3).

#### 3.6.3. Kidney Histopathology

**Figure 4:**
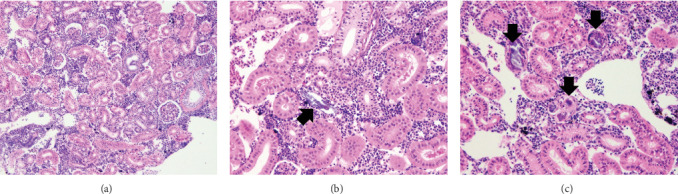
Photomicrograph (H&E, 20× magnification) of normal posterior kidney representative of Diets 1–3 (a) in contrast with photomicrographs of posterior kidney representative of Diet 4 (b) showing mineral surrounded by fibrous connective tissue (arrow) and Diet 5 (c) showing mineral replacing the parenchyma (arrows).

**Figure 5:**
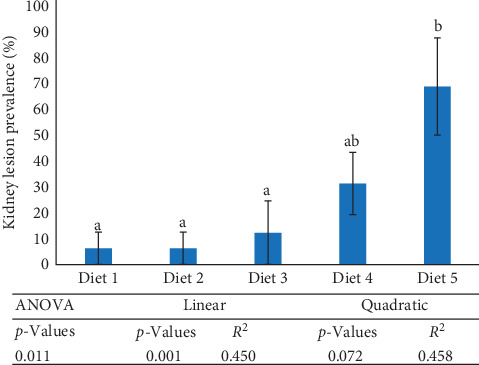
Kidney lesion prevalence (%) of rainbow trout fed experimental diets for 13 weeks. Data represent means ± SEM (*n* = 16 fish per treatment). Means with different letters are significantly different (*p*  < 0.05; ANOVA with post hoc Tukey's HSD). ANOVA, analysis of variance.

The general conformation of the kidney for the rainbow trout was within normal limits (Figure 4). In the kidney, all samples had intact renal tubules, collecting ducts, vessels (arteries, capillaries, veins), and glomeruli supported by a fine fibrovascular stroma (Figure 4). Adequate hematopoietic tissues expanded the interstitium between tubules. Similar to the liver, there was minimal-to-mild pathology associated with the parenchyma. Although all diets were affected by rare mineralization, Diet 5 has significantly more fish (*n* = 10 among *n* = 16) noted with pathology than all other groups. Lesions were characterized by small amounts of mineralized basophilic amorphous material filling or replacing tubules (Figure 4). In sections where the mineral had replaced tubules, a thin layer of fibrous connective tissue surrounded the amorphous mineral fragments (Figure 4). The second most affected group was Diet 4, with five fish having rare foci of mineralization. Fish fed Diet 5 had a kidney lesion prevalence that was considerably greater than fish fed Diet 1, Diet 2, and Diet 3, while fish received Diet 4 had an intermediate lesion prevalence (Figure 5). Across treatments, kidney lesion prevalence (%) followed a linear trend but not a quadratic trend (*p*  > 0.05).

## 4. Discussion

Protein meals produced from insects have drawn substantial attention as novel feed ingredients for aquafeeds since they contain a high amount of protein and potential functional properties for supporting fish growth and health. Even so, high lipid content in insect meals often works as a barrier for high inclusion rates in aquafeeds due to the difficulty of processing during feed manufacturing. To overcome this limitation, insect meal producers have recently expanded the output of defatted insect meal, which has higher protein and a lower lipid content. Mealworms are one of the most significant insect species that the EU has approved, and they have been effectively employed as a partial substitution for traditional protein sources. However, in most of the research conducted in fish, especially in salmonids, DMW meal inclusion has been restricted to ≤20% of the diet [12]. Higher inclusion of mealworm meals may be desirable as supplies increase to meet the shift toward more sustainable ingredients in aquafeeds. Hence, a higher inclusion (up to 40%) of DMW meal has been assessed in this study as a total FM replacer in rainbow trout diets.

In the current research, the complete substitution of FM with DMW meal caused no discernable differences in the growth performance of rainbow trout. These findings indicate that DMW meal could be used to completely replace FM. In previous research on rainbow trout, Belforti et al. [17] reported that 33%–67% substitution of FM with full-fat mealworm meal prompted an improvement in growth performance. Chemello et al. [12] also reported a total substitution of FM with defatted yellow mealworm meal in grower diets of rainbow trout without any adverse effects on growth parameters. These results are comparable to those of our study when the percentage of FM replacement is being considered. However, in terms of DMW inclusion level, the latter study [12] included a maximum of 20% DMW meal, which is substantially lower than our study, where a maximum of 40% DMW meal was used in the diet. In other fish species, like red sea bream, a higher inclusion (a maximum of 65%) of DMW meal as a complete FM replacer has been reported [11]. In contrast, a relatively lower tolerance of DMW meal inclusion has been reported for other species. European perch *Perca fluviatilis* can tolerate 6.8% (25% FM replacement) [30], large yellow croakers can tolerate 13% (30% FM substitution) [13], and African catfish *Clarias gariepinus* can tolerate 17.3% (40% FM replacement) [31] without negatively affecting growth. The variations in these findings could be attributed to various aspects of FM, including its source and quality, overall diet composition, the amount of DMW meal added, fish species-related differences in digestive enzymes, raw material processing technology, and other factors.

In the present investigation, it was found that replacing FM with DMW had no appreciable impact on feed intake or feed utilization metrics. However, there was a tendency toward increased feed intake and lower feed efficiency that, over a longer growth period, could impact the economics of production depending on the pricing of DMW meal relative to other protein feedstuffs. Higher feed intake might be associated with the functional compounds present in the DMW meal. Reduced feed efficiency, however, has been linked with the existence of high chitin content in insect meals [32–34]. In a previous study, Kroeckel et al. [33] ascertained that rainbow trout given diets with 10% chitin inclusion showed decreased feed utilization performance. In addition, Karlsen et al. [35] noticed decreased growth rate and apparent protein digestibility when chitin concentration rose in salmon diets from 1% to 2% and 5%. In the present study, the amount of chitin was not determined.

The rate at which nutrients from the feed are used by organisms can be indicated by the apparent digestibility coefficient of conventional nutrients. In the current study, DMW meal apparent digestibility of dry matter, protein, lipid, ash, energy, and phosphorous in rainbow trout were 79.49%, 87.42%, 105.18%, 106.43%, 84.2%, and 90.0%, respectively. The DMW meal digestibility findings in our investigation align with the findings of Basto, Matos, and Valente [36], where dry matter, protein, lipid, energy, and phosphorous digestibility were 72.4%, 92.8%, 94.4%, 84.4%, and 90.7%, respectively, in European sea bass. Also, according to Fontes et al. [37], dry matter, protein, lipid, and energy digestibility in Nile tilapia were 95.8%, 85.4%, 90.6%, and 82.1%, respectively. Meanwhile, it is critically important to look at the amino acid digestibility of protein sources because protein digestibility is directly related to amino acid digestibility. The current study found that the majority of the amino acid digestibility coefficients for DMW meal were >90%, except for cysteine (79.30%) and taurine (72.34%). The obtained amino acid digestibilities of DMW meal are almost in line with those obtained by Basto, Matos, and Valente [36] in European sea bass. In comparison to the DMW meal apparent digestibility coefficients of macronutrients and amino acids for rainbow trout, other conventional protein feedstuffs, like FM and poultry meal, are nearly similar and higher than plant protein ingredients such as corn and soy protein concentrates [38]. Taken together, these results suggest that the DMW meal digestibility is influenced by species, growth stage, and the processing technology, but that DMW meal can be efficiently digested and absorbed by rainbow trout.

In this study, DMW inclusion as an FM replacer significantly influenced the whole-body moisture content. Fish-fed control and Diet 5 showed significantly higher and lower moisture content, respectively. In contrast, whole-body lipids showed an opposite trend. An inverse correlation between fish body moisture and fat content was observed, as reported previously in fish [39–41]. Significantly higher whole-body lipid contents in DMW meal included groups may reflect the high lipid digestibility of DMW meal. However, fillet proximate compositions in this study were not significantly impacted by DMW incorporation as an FM replacer. The composition of rainbow trout fillets following an insect feed growth trial has been assessed in several research studies [17, 42, 43]. Compared with our findings, some of the previous studies found no significant influence of DMW meal inclusion as an FM replacer on fillet proximate composition [44–46]. Meanwhile, others described a small decrease in crude protein or lipid content in the fillet of insect meal-fed fish [17, 42, 47]. These discrepancies could result from the fish size, types, and levels of insect meal utilized in these studies.

Hematological and arterial blood characteristics are employed as an effective and sensitive indicator of fish wellbeing as well as physiological and pathological conditions [48]. The measured hematological and arterial blood parameters, blood gases, and electrolytes often provide an important clue for the wellbeing of many vital organs, including the heart, skeletal muscle, intestine, and the lungs for higher vertebrates, with similar effects for poikilothermic fish [49]. The use of DMW meal as an FM substitute did not show any significant alteration of measured hematological electrolyte, acid–base, and blood gas values, except for sodium (Na) concentration. Although fish given Diets 4 and 5 had significantly higher values of Na concentrations, these values remain within the normal range reported for blood Na concentration of rainbow trout (139.00–168.00 mmol/L) [50]. The lack of influence on PO_2_, pCO_2_, TCO_2_, ionized calcium, sO_2_, hematocrit, and hemoglobin with DMW meal inclusion indicates that DMW meal does not cause adverse effects on blood gas pressure and electrolyte balance in rainbow trout. Further, these results suggest comparable functionality of gill and kidney of fish-fed DMW versus fish-fed FM-based diets.

Assessment of tissue morphology and pathology of different digestive organs through histological analysis is a widely accepted method to evaluate the efficiency of using new diets having alternative protein ingredients. In the present study, distal intestine, liver, and kidney tissues were assessed through histological procedure to assess the efficiency of using DMW meal as an FM alternative in rainbow trout. In the case of the intestinal histopathology assessment, indices for intestinal enteritis were similar across treatment groups, with only LP thickness being significantly influenced by DMW meal inclusion. Significantly greater LP thickness was noticed in DMW included diet groups in comparison to the FM-based control group. The LP's thickness is typically correlated with the quantity of lymphoid cells present and the area's particular function [51]. It is hypothesized that the thickness of the LP increases to allow ample room for the lymphoid cells. As DMW meal inclusion increased, there were higher concentrations of lymphoid cells in the thicker LP area, which may indicate that these diet groups had an immunostimulatory effect [52, 53]. Although in our study, immunomodulatory effects of DMW meal were not evaluated, it is well established from previous studies that dietary mealworm meal may have immunostimulatory functions in fish [54–56] because of the presence of several functional materials, like chitosan (produced from chitin through deacetylation), antimicrobial peptides, and antifreeze protein in mealworm meal [9]. As a measure of gross distal intestinal enteritis, the mean histopathological score did not significantly vary from the FM-based control diet group until 75% of the FM was substituted with DMW. A significantly higher mean score in the complete FM-replaced group with DMW indicates some degrees of intestinal enteritis; however, scores <2 indicate minor morphological changes and are not consistent with intestinal inflammation.

The liver is the primary site for various important physiological functions in fish, such as protein production, bile production, blood filtration, regulation of amino acids, production of key coagulation components, filtration of pathogens, storage of vitamins/minerals, and glucose processing [57]. Dietary modification/inclusion of new ingredients can influence the structure and function of this vital organ. In this study, all treatment groups showed mild inflammation in the liver. However, significantly lower liver inflammation was observed in fish-fed Diet 3. On the other hand, Diet 5 (100% FM replacement with DMW meal) had the highest level of inflammation. Other studies have also shown histopathological changes in fish liver given insect meals, particularly at high inclusion levels; examples include in largemouth bass *Micropterus salmoides* given diets containing high dietary mealworm meal [58], in red hybrid tilapia *Oreochromis sp*. consuming feeds containing house cricket *Acheta domesticus* meal in graded levels [59], and in Jian carp *Cyprinus carpio* var. Jian consuming diets with high amounts of defatted black soldier fly meal [60]. In this study, the vacuolar change noted in the liver of several fish from each treatment group is not considered pathogenic as it is most often linked with age, reproductive hormones, nutrient storage, and life stage of the fish. However, the reduction in inflammation observed with moderate dietary DMW meal inclusion (Diet 3) suggests improved liver health and a possible functional benefit. To understand this effect, further investigation is warranted.

In the kidney, a different trend was observed. The prevalence of lesions which were characterized by small amounts of mineralized basophilic amorphous material filling or replacing tubules, was considerably higher in fish given Diet 5 relative to other dietary groups. In various fish species, including rainbow trout, renal mineralization (nephrolithiasis) has been documented, but the pathogenesis remains unclear [61, 62]. Mineral deposition within the kidney is considered a multifactorial process, with diet, environmental conditions, and metabolic components all playing an important role. Nephrolithiasis in fish has been attributed to prolonged CO_2_ exposure, selenium toxicity, magnesium deficiency, and unbalanced mineral content within feed or culture environments [63]. However, these explanations are unlikely with the water source, culture system and dietary mineral premix used in the current study. For now, the mechanism of action remains unclear and requires further investigation.

## 5. Summary and Conclusion

In summary, rainbow trout growth and feed utilization were not adversely impacted by the total replacement of FM with DMW meal in the diet, although 100% FM replacement and 40% DMW meal inclusion resulted in greater whole-body lipid content. DMW meal apparent digestibility coefficients for major nutrients and amino acids are comparable with the digestibility of FM and black solder fly meal. Complete FM replacement with DMW meal also had little to no effect on blood gas pressure, electrolyte balance, and hematocrit. Although all dietary groups displayed some intestinal enteritis, none were severe. In fact, the 75% FM replacement with the DMW meal group showed significantly lower intestinal enteritis. Histology of liver and kidney tissue samples indicated a very low level of lymphocytic inflammation in the liver and mineralization in the posterior kidney within all groups; however, the 50% FM replacement with DMW meal improved liver health with no negative impact on kidney histopathology. Finally, considering the study's overall results, the conclusion can be drawn that the DMW meal examined can be utilized successfully as an FM replacement in the diets of rainbow trout and that optimal inclusion levels are between 50% and 75% FM replacement or 20% and 30% dietary inclusion.

## Figures and Tables

**Table 1:** Proximate composition (as-is bases) of defatted mealworm (DMW) meal (MidWest Laboratories, Omaha, Nebraska).

Parameters	Compositions (%)
Moisture	4.2
Crude protein	72.4
Crude fat	5.3
Fiber (acid detergent)	11.4
Ash	6.6

**Table 2:** Formulation and proximate composition of the experimental diets fed to rainbow trout for 13 weeks.

Ingredients	Diets				
	Diet 1	Diet 2	Diet 3	Diet 4	Diet 5
Fish meal, sardine*⁣*^*∗*^	40.00	30.00	20.00	10.00	0.00
DMW meal^†^	0.00	10.00	20.00	30.00	40.00
Soybean meal*⁣*^*∗*^	9.50	11.50	10.00	8.00	6.00
Wheat gluten meal*⁣*^*∗*^	7.00	7.00	7.00	7.00	7.00
CPC^§^	10.00	8.00	7.00	6.00	5.00
L-lysine HCl^¶^	0.00	0.00	0.53	1.00	1.65
DL-methionine^¶^	0.00	0.00	0.00	0.12	0.23
Wheat flour*⁣*^*∗*^	16.50	15.38	16.45	17.86	19.20
Dicalcium phosphate*⁣*^*∗*^	0.00	1.42	2.52	3.62	4.72
Trace mineral mix, Trouw^#^	0.10	0.10	0.10	0.10	0.10
Vitamin Premix, ARS^††^	1.00	1.00	1.00	1.00	1.00
Choline chloride (60%)*⁣*^*∗*^	0.60	0.60	0.60	0.60	0.60
Stay C (35%) vitamin*⁣*^*∗*^	0.20	0.20	0.20	0.20	0.20
Fish oil^‡^	15.10	14.80	14.60	14.50	14.30
Total	100	100	100	100	100

*⁣* ^*∗*^Rangen Inc., Buhl, ID, USA.

^†^DMW (defatted meal worm); Beta Hatch (Cashmere, WA, USA).

^§^CPC (corn protein concentrate); Empyreal 75, Cargill Corn Milling, Blair, NE, USA.

^¶^Sigma Aldrich, St. Louis MQ, USA.

#US Fish and Wildlife Service Trace Mineral Premix #3. It supplied the following (mg/kg diet): Zn (as ZnSO_4_.7H_2_O), 75; Mn (as MnSO_4_), 20; Cu (as CuSO_4_.5H_2_O), 1.54; I (as KIO_3_), 10.

^††^Vitamin premix supplied the following per kg diet: vitamin A, 2.4 mg; vitamin D, 0.15 mg; vitamin E, 267 mg; vitamin K as menadione sodium bisulfite, 20 µg; thiamin as thiamin mononitrate, 32 mg; riboflavin, 64 mg; pyridoxine as pyridoxine-HCl, 64 mg; pantothenic acid as Ca-d-pantothenate, 192 mg; niacin as nicotinic acid, 240 mg; biotin, 0.56 mg; folic acid, 12 mg; vitamin B_12_, 50 µg; and inositol as meso-inositol, 400 mg.

^‡^Skretting USA, Tooele, UT, USA.

**Table 3:** Ingredient composition of reference mash (%, as-fed basis) used for ingredient digestibility study.

Ingredient	Inclusion level
Fishmeal, sardine*⁣*^*∗*^	33.00
Soy protein concentrate^†^	13.90
Corn protein concentrate^§^	10.00
Wheat flour*⁣*^*∗*^	18.00
Wheat gluten meal*⁣*^*∗*^	7.10
Dicalcium phosphate*⁣*^*∗*^	1.20
Choline chloride (60%)*⁣*^*∗*^	0.60
Vitamin C (Stay C, 35%)*⁣*^*∗*^	0.20
Vitamin premix, ARS 702^¶^	0.80
Trace mineral mix, Trouw^#^	0.10
Fish oil, Alaska pollock^††^	15.00
Yttrium oxide^‡^	0.10
Total	100

*⁣* ^*∗*^Rangen Inc., Buhl, ID, USA.

^†^Profine VF, The Solae Company, St. Louis, MO, USA.

^§^Empyreal 75, Cargill Corn Milling, Cargill, Inc., Blair, NE, USA.

^¶^Vitamin premix supplied the following per kg diet: vitamin A, 2.4 mg; vitamin D, 0.15 mg; vitamin E, 267 mg; vitamin K as menadione sodium bisulfite, 20 µg; thiamin as thiamin mononitrate, 32 mg; riboflavin, 64 mg; pyridoxine as pyridoxine-HCl, 64 mg; pantothenic acid as Ca-d-pantothenate, 192 mg; niacin as nicotinic acid, 240 mg; biotin, 0.56 mg; folic acid, 12 mg; vitamin B_12_, 50 µg; and inositol as meso-inositol, 400 mg.

#US Fish and Wildlife Service Trace Mineral Premix #3 supplied the following (mg kg−1 diet): Zn (as ZnSO_4_·7H_2_O), 75; Mn (as MnSO_4_), 20; Cu (as CuSO_4_·5H_2_O), 1.54; I (as KIO_3_), 10.

^††^Skretting USA, Tooele, UT, USA.

^‡^Sigma Aldrich, St. Louis MQ, USA.

**Table 4:** Analyzed major nutrients and amino acids composition of experimental diets (%, as-fed basis).

Parameters*⁣*^*∗*^	Diets					
	Diet 1		Diet 2	Diet 3	Diet 4	Diet 5
Moisture (%)	4.94	4.78		4.83	5.07	4.88
Crude protein (%)	47.02	47.11		46.48	47.42	47.10
Crude lipid (%)	16.81	16.01		17.42	16.02	16.06
Ash (%)	5.48	5.56		5.65	5.71	5.71
Energy (MJ/g)	22.23	22.21		22.35	22.27	22.67
Alanine	2.58	2.60		2.62	2.71	2.71
Arginine	2.61	2.51		2.41	2.34	2.23
Aspartic acid	4.22	4.05		3.83	3.72	3.50
Cysteine	0.64	0.60		0.57	0.55	0.52
Glutamic acid	8.01	7.66		7.36	7.00	6.53
Glycine	1.85	1.87		1.88	1.96	1.97
Histidine	1.05	1.07		1.09	1.17	1.20
Isoleucine	2.27	2.20		2.08	2.07	2.01
Leucine	4.17	4.05		3.88	3.84	3.67
Lysine	4.28	4.25		4.16	4.23	4.16
Methionine	1.40	1.36		1.36	1.38	1.35
Phenylalanine	2.21	2.16		2.07	2.06	1.96
Proline	2.32	2.45		2.56	2.75	2.84
Serine	1.78	1.78		1.86	1.78	1.63
Taurine^†^	0.21	0.16		0.15	0.14	0.14
Threonine	2.28	2.34		2.37	2.43	2.37
Tryptophan	0.49	0.49		0.48	0.49	0.49
Tyrosine	1.63	1.81		2.10	2.30	2.48
Valine	2.42	2.41		2.38	2.46	2.51

*⁣* ^*∗*^Results are expressed on an “as is” basis unless otherwise indicated.

^†^Nonproteinogenic amino acids.

**Table 5:** Growth performance and feed utilization of rainbow trout fed with experimental diets for 13 weeks.

Variables*⁣*^*∗*^	Diet groups					SEM^†^	ANOVA	Linear	*R* ^2^	Quadratic	*R* ^2^
	Diet 1	Diet 2	Diet 3	Diet 4	Diet 5		*p*-Values	*p*-Values		*p*-Values	
IW	23.1	23.5	23.4	23.0	23.2	0.4	—	—	—	—	—
FW	264.4	242.4	268.3	264.6	264.9	10.1	0.404	0.475	0.028	0.697	0.033
WG	1048.6	932.5	1049.0	1051.2	1040.8	51.5	0.438	0.537	0.021	0.622	0.052
SGR	2.68	2.56	2.68	2.68	2.68	0.052	0.399	0.507	0.024	0.591	0.047
FI	171.8	166.5	187.0	191.2	186.4	9.3	0.299	0.086	0.166	0.668	0.170
FCR	0.72	0.76	0.76	0.79	0.77	0.026	0.351	0.088	0.168	0.321	0.183
PER	3.00	2.80	2.82	2.68	2.75	0.10	0.294	0.076	0.178	0.329	0.185
SUR	94.4	86.9	91.9	87.5	96.9	3.9	0.350	0.658	0.010	0.119	0.118
CF	1.59	1.56	1.53	1.59	1.53	0.056	0.855	0.636	0.014	0.909	0.056
HSI	1.21	1.15	1.15	1.19	1.07	0.048	0.326	0.123	0.133	0.745	0.151

*⁣* ^*∗*^CF, condition factor = weight of fish/(length of fish)^3^ × 100; FCR, feed conversion ratio; FI, feed intake (g fish^−1^); FW, final weight (g); HSI, hepatosomatic index (%) = weight of liver/weight of fish × 100; IW, initial weight (g); PER, protein efficiency ratio; SGR, specific growth rate (% day^−1^); SUR, survival; WG, weight gain (%).

^†^Standard error of the mean.

**Table 6:** Digestibility coefficients (%) of defatted mealworm (DMW) meal fed to rainbow trout.

Variables*⁣*^*∗*^	Digestibility (%)
ADC_DM_	79.49
ADC_protein_	87.42
ADC_lipid_	105.18
ADC_ash_	106.43
ADC_energy_	84.20
ADC_phosphorous_	90.00
Alanine (Ala)	92.65
Arginine (Arg)	100.05
Aspartate (Asp)	92.79
Cysteine (Cys)	79.30
Glutamate (Glu)	96.39
Glycine (Gly)	91.80
Histidine (His)	94.51
Isoleucine (Ile)	95.31
Leucine (Leu)	95.05
Lysine (Lys)	108.06
Methionine (Met)	93.11
Phenylalanine (Phe)	93.38
Proline (Pro)	94.27
Serine (Ser)	91.25
Taurine (Tau)	72.34
Threonine (Thr)	94.41
Tryptophan (Trp)	102.86
Tyrosine (Tyr)	95.07
Valine (Val)	96.42

*⁣* ^*∗*^Apparent digestibility coefficient (ADC).

**Table 7:** Whole body and muscle proximate analysis (% wet basis) of rainbow trout fed the test diets for 13 weeks.

Parameters	Initial*⁣*^*∗*^	Diet groups						SEM^†^	ANOVA	Linear	*R* ^2^	Quadratic	*R* ^2^
		Diet 1	Diet 2	Diet 3	Diet 4		Diet 5		*p*-Values	*p*-Values		*p*-Values	
Whole-body proximate analysis													
Moisture	76.38	68.81^b^	67.66^a,b^	67.63^a,b^	67.49^a,b^		66.70^a^	0.393	0.029	0.003	0.418	0.684	0.425
Protein	14.83	16.57^a,b^	16.72^a,b^	16.44^a,b^	17.13^b^		16.37^a^	0.164	0.036	0.977	2.909	0.185	0.048
Lipid	5.5	12.38^a^	13.70^a,b^	13.62^a,b^	13.45^a,b^		14.83^b^	0.486	0.044	0.009	0.326	0.986	0.349
Ash	2.47	1.94	1.93	1.83	1.94		1.96	0.067	0.700	0.861	0.002	0.292	0.044
Energy^§^	24.50	28.8^a^	28.79^a,b^	28.67^a,b^	28.71^a,b^		29.41^b^	0.22	0.031	0.006	0.347	0.511	0.347
Muscle proximate analysis													
Moisture	—	73.37	73.66	73.35	72.57		73.20	0.297	0.168	0.146	0.104	0.854	0.129
Protein	—	20.33	20.54	20.55	20.42		20.58	0.166	0.794	0.466	0.033	0.696	0.033
Lipid	—	5.16	4.83	5.12	5.72		5.23	0.307	0.387	0.311	0.057	0.992	0.058
Ash	—	2.07	2.09	2.21	2.00		2.17	0.056	0.145	0.553	0.019	0.938	0.027
Energy^§^	—	25.60	25.51	25.64	25.73		25.48	0.16	0.814	0.946	2.852	0.582	0.019

*⁣* ^*∗*^Initial values are not included in the statistical analysis.

^†^Standard error of the mean.

^§^Energy (MJl/kg).

^a,b^Means with different letters are significantly different (*p*  < 0.05; ANOVA with post hoc Tukey's HSD).

**Table 8:** Hematological and arterial blood parameters (electrolyte, acid–base, and blood gas values) of rainbow trout fed with experimental diets for 13 weeks.

Variables*⁣*^*∗*^	Diet groups					SEM^†^	ANOVA	Linear	*R* ^2^	Quadratic	*R* ^2^
	Diet 1	Diet 2	Diet 3	Diet 4	Diet 5		*p*-Values	*p*-Values		*p*-Values	
pH	6.88	6.88	6.88	6.82	6.89	0.015	0.044	0.333	0.036	0.171	0.039
PCO_2_	40.34	41.01	40.66	42.51	42.74	1.243	0.553	0.130	0.142	0.779	0.156
PO_2_	7.63	8.06	8.69	8.06	8.63	0.706	0.809	0.384	0.048	0.710	0.080
BEcef	−25.5	−25.5	−25.69	−25.94	−24.94	0.424	0.570	0.615	0.015	0.240	0.230
HCO_3_	7.62	7.68	7.54	7.65	8.18	0.305	0.606	0.275	0.072	0.317	0.202
TCO_2_	8.75	8.88	8.88	9.06	9.56	0.319	0.432	0.092	0.170	0.444	0.231
sO_2_	3.44	3.50	3.88	3.63	3.81	0.447	0.944	0.545	0.024	0.825	0.045
Na	149.50^a^	149.69^a^	150.94^a,b^	149.56^a^	152.25^b^	0.527	0.008	0.006	0.292	0.247	0.346
K	2.36	2.25	2.26	2.11	2.02	0.149	0.509	0.093	0.174	0.800	0.237
iCa	1.63	1.64	1.65	1.66	12.51	4.840	0.438	0.175	0.106	0.250	0.404
Glu	67.06	71.13	68.19	67.44	68.06	3.178	0.902	0.869	0.002	0.699	0.325
Ht	34.38	37.0	36.19	36.75	38.88	0.169	0.480	0.122	0.144	0.953	0.158
Hb	11.69	12.58	12.30	12.49	13.23	0.571	0.477	0.122	0.144	0.941	0.159

*⁣* ^*∗*^BEcef, base excess (mol/L); Glu, glucose (mg/dL); Hb, hemoglobin (g/dL); HCO_3_, bicarbonate (mmol/L); Ht, hematocrit (%PCV); iCa, ionized calcium (mmol/L); K, potassium (mmol/L); Na, sodium (mmol/L); pCO_2_, partial pressure of carbon dioxide (mmHg); pH, acidity; PO_2_, partial pressure of oxygen (mmHg); sO_2_, oxygen saturation (%); and TCO_2_, total carbon dioxide (mmol/L).

^†^Standard error of the mean.

^a,b^Means with different letters are significantly different (*p*  < 0.05; ANOVA with post-hoc Tukey's HSD).

**Table 9:** Histopathology of the distal intestine of rainbow trout fed experimental diets for 13 weeks.

Parameters*⁣*^*∗*^	Diet groups					SEM^†^	ANOVA	Linear	*R* ^2^	Quadratic	*R* ^2^
	Diet 1	Diet 2	Diet 3	Diet 4	Diet 5		*p*-Values	*p*-Values		*p*-Values	
MFF	1.19	1.19	1.19	1.13	1.38	0.093	0.417	0.303	0.059	0.226	0.098
GC	1.06	1.13	1.19	1.00	1.19	0.074	0.346	0.601	0.014	1.00	0.114
SV	1.19	1.13	1.19	1.00	1.25	0.094	0.431	1.00	0.000	0.304	0.240
EG	1.06	1.13	1.38	1.13	1.19	0.094	0.218	0.414	0.033	0.176	0.045
LP	1.00	1.13	1.25	1.13	1.44	0.095	0.053	0.011	0.311	0.731	0.315
SeM	1.13	1.34	1.19	1.33	1.31	0.108	0.540	0.312	0.060	0.681	0.182
Mean score	1.11	1.18	1.23	1.12	1.29	0.049	0.084	0.058	0.168	0.813	0.170

*⁣* ^*∗*^EG, eosinophilic granulocyte; GC, goblet cell; LP, lamina Propria; MFF, mucosal fold fusion; SeM, subepithelial mucosa; SV, supranuclear vacuole.

^†^Standard error of the mean.

## Data Availability

The data that support the findings of this study are available from Beta Hatch. Restrictions apply to the availability of these data, which were used under license for this study. Data are available from the author(s) with the permission of Beta Hatch.
